# Changes in Sensorimotor Cortical Activation in Children Using Prostheses and Prosthetic Simulators

**DOI:** 10.3390/brainsci11080991

**Published:** 2021-07-27

**Authors:** Christopher Copeland, Mukul Mukherjee, Yingying Wang, Kaitlin Fraser, Jorge M. Zuniga

**Affiliations:** 1 Department of Biomechanics, University of Nebraska-Omaha, Omaha, NE 68182, USA; ccopeland@unomaha.edu (C.C.); mmukherjee@unomaha.edu (M.M.); kfraser@unomaha.edu (K.F.); 2 Department of Special Education and Communication Disorders, University of Nebraska-Lincoln, Lincoln, NE 68583, USA; yingying.wang@unl.edu

**Keywords:** prosthetic simulator, upper limb reduction, motor cortex, somatosensory cortex, fNIRS

## Abstract

This study aimed to examine the neural responses of children using prostheses and prosthetic simulators to better elucidate the emulation abilities of the simulators. We utilized functional near-infrared spectroscopy (fNIRS) to evaluate the neural response in five children with a congenital upper limb reduction (ULR) using a body-powered prosthesis to complete a 60 s gross motor dexterity task. The ULR group was matched with five typically developing children (TD) using their non-preferred hand and a prosthetic simulator on the same hand. The ULR group had lower activation within the primary motor cortex (M1) and supplementary motor area (SMA) compared to the TD group, but nonsignificant differences in the primary somatosensory area (S1). Compared to using their non-preferred hand, the TD group exhibited significantly higher action in S1 when using the simulator, but nonsignificant differences in M1 and SMA. The non-significant differences in S1 activation between groups and the increased activation evoked by the simulator’s use may suggest rapid changes in feedback prioritization during tool use. We suggest that prosthetic simulators may elicit increased reliance on proprioceptive and tactile feedback during motor tasks. This knowledge may help to develop future prosthesis rehabilitative training or the improvement of tool-based skills.

## 1. Introduction

### 1.1. Incidence of Limb Reduction

Over two million people are currently living with some sort of limb loss in the United States alone [1]. Additionally, 1 in 1900 children is born with an impairment known as congenital limb reduction deficiency each year [2], which results in variable levels of amputation, from finger and toe reduction to complete removal of the limb. Motor development is often blunted in these populations due to a lack of rehabilitation through the early fitting of a prosthesis, which may cause functional detriments in their daily life [3,4,5]. Presently, there is a deficiency in motor research in children with congenital upper limb reduction (ULR) due to the scarce availability of research participants [6,7]. This deficiency in research participants has created a gap in the literature regarding the immediate effects and adaptation of congenital loss or early traumatic loss of a limb on the brain and its body schema [8,9].

### 1.2. Body Schema and Phantom Sensations

The body schema refers to the internal representation of the body within the brain and appears to be altered in individuals with ULR [9,10]. This schema supplies the brain with information such as limb size and position, and is used in the planning of movements [11]. Mail-in survey studies have reported that anywhere from 8 to 18% of congenital amputees experience phantom limb sensations [8,12]. The low incidence of phantom sensations within individuals with congenital reductions raises the question of whether the body schema is innate or acquired through experience. Further studies suggest that the schema of the affected limb of individuals with congenital reductions is either missing or dormant. Congenital amputees appear to lack the cortical reorganization present in traumatic amputees with phantom limb pain [10]. Furthermore, using transcranial magnetic stimulation over the site of the affected limb has been unsuccessful in eliciting any phantom sensations [9]. These observations continue to drive the question of whether body schema is innate or driven by experience.

### 1.3. Neuronal Group Selection Theory and Regions of Interest

The Neuronal Group Selection Theory (NGST) is a theoretical framework used to describe plasticity within the brain, which drives differential cortical activations [13,14]. NGST suggests that neurons are organized into various neuronal networks or groups, which can be dynamically altered through innate development and behavior. Specifically, from birth until roughly one year of age, children are in a state of developmental selection. In this stage, the selection of neuronal groups is driven by epigenetic variation that alters synaptic networks. After one year of age, children enter the secondary stage, or experiential selection. During experiential selection, secondary groups are produced by specific patterns of behavior-driven activation. Groups that are selected have amplified synaptic strength, while neglected groups are weakened. As such, it stands to reason that children with ULR have reduced ability to create secondary groups through experiential selection in the somatotopic regions for their affected limb, due to the physical constraints imposed by their reduction [14,15,16]. In this study, we have chosen to observe neural activation within the primary motor cortex (M1), and the primary somatosensory cortex (S1) due to their direct somatotopy [17,18], as well as the supplementary motor area (SMA) due to its involvement in motor planning and complex or repetitive movements [19].

### 1.4. Prosthetic Simulators 

Prosthetic simulators are a novel tool used within rehabilitation and research to emulate motor control conditions of a prosthetic device using an unaffected limb. They have the potential to aid amputees through cross-education, in which experience with the simulator using the non-affected hand improves motor function with their prosthetic device [20,21]. These devices limit distal effectors such as the fingers and force the user to utilize the terminal grasping device of the simulator. These tools can be used by a wide population and can thus be used to expedite the development of new prosthetic rehabilitation paradigms translated into clinical populations [20]. Studies involving prosthetic simulators have observed the kinematic and functional results from the effects of variability of practice [22] and from specific training paradigms [6,23,24]. These studies have suggested simulators to be useful tools for improving prosthetic rehabilitation through cross-education [6,7,23,24,25]. However, it is unclear whether prosthetic simulators can induce similar neurological adaptations seen in novice prosthesis users. The ability to emulate such adaptations would aid in elucidating the neural bases of prosthetic learning.

### 1.5. Functional Near-Infrared Spectroscopy

Functional near-infrared spectroscopy (fNIRS) is a rapidly progressing tool for the non-invasive investigation of cortical activity during functional tasks [26]. This measurement is based on neurovascular coupling, where the fNIRS measures the changes in the brain tissue concentration of oxygenated hemoglobin (HbO) and deoxygenated hemoglobin (HbR) as neuronal activity creates increased metabolic demands that require more oxygen perfusion to the tissue [27]. Due to its non-invasive and flexible nature, many studies have successfully implemented fNIRS systems in their studies that investigate pediatric populations [28,29,30].

### 1.6. Aims and Hypothesis

The aim of the present study was to investigate differences in the contralateral hemodynamic activity of children with ULR using a prosthesis and typically developing (TD) children using a prosthetic simulator. Furthermore, we sought to determine the effect of the prosthetic simulator on contralateral hemodynamic response during a gross motor task in TD children. We hypothesized that the neural response in children with ULR using their prosthesis would be lower across all regions compared to TD children using a prosthetic simulator due to weakened neuronal groups. We additionally hypothesized that use of the simulator in the TD group would increase contralateral activation compared to use of the non-preferred hand as the novelty of the tool would force the selection of new neuronal groups.

## 2. Materials and Methods

### 2.1. Participants 

Five children with left unilateral ULR (mean age: 8.76 ± 3.37 years, 3 male and 2 female, reported to be right-handed) and five age-, sex-, and handedness-matched TD children (mean age: 8.96 ± 3.23) participated in this study. See Table 1 for full participant characteristics. Inclusion criteria for the ULR group included boys and girls from 6 to 13 years of age with unilateral carpus or radial upper limb reduction who had not used a prosthesis within the past 6 months. The inclusion criteria for the TD group included boys and girls aged 6 to 13 years. Exclusion criteria for both groups included upper extremity injury within the past month or medical conditions that are contraindications for wearing a prosthesis or a prosthetic simulator (such as skin abrasions and musculoskeletal injuries in the upper limbs). The study was approved by the Institutional Review Board of the University of Nebraska Medical Center. An assent was explained by the corresponding author and signed by the children and their parents. Both parents and children were given an overview of the experiment before the trials began.

### 2.2. Experimental Design

Hemodynamic responses in the contralateral M1, SMA, and S1 were recorded for three one-minute trials of the Box and Block test [31], with one minute of rest between trials. The ULR group performed the task with their prosthesis, while the TD group used their non-preferred hand, with and without a prosthetic simulator.

The Box and Block test is a measure of unilateral gross dexterity, and normative data have been collected using it for both adults and typically developing children [32,33]. The Box and Block test involves moving 2.5cm^3^ blocks from one side of a 53.7 cm × 25.4 cm × 8.5 cm partitioned box to the other. Before testing began, the children were provided with instructions and a demonstration of the task. After a 15 s practice period, the children were then asked to place their hands on the side of the box. During the task, the children moved one block at a time, transporting it over the partition and dropping it in the opposite compartment. The children were instructed to move as many blocks as possible within 60 s. This was followed by 60 seconds of rest. Three trials were performed for each limb condition (prosthetic and preferred for ULR, simulator and non-preferred for TD). The number of blocks moved was recorded for each trial. If two or more blocks were moved at the same time, this was noted and counted as one block. Blocks that fell outside of the box were not counted.

### 2.3. Prostheses and Prosthetic Simulators 

**Figure 1:**
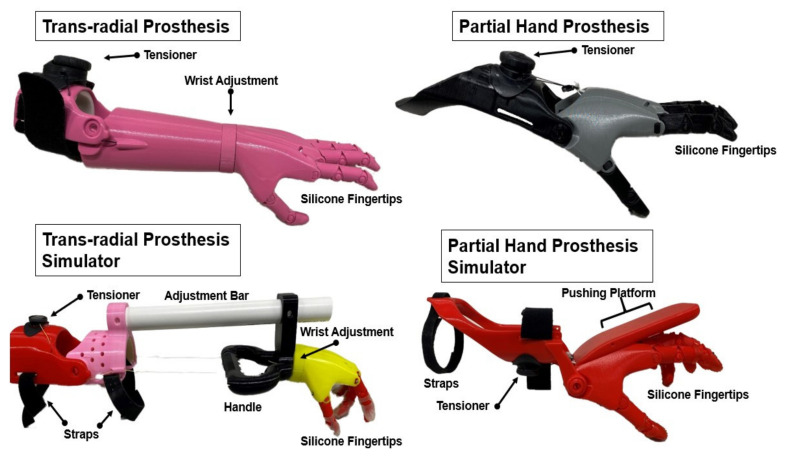
Description of prostheses and prosthetic simulators. The prosthetic simulators used in the study mimic the design and control mechanism of the prostheses. The partial hand prosthesis simulator allowed TD children to rest their existing hand on top of the simulator hand, with the wrist in slight extension. A pushing platform placed above the hand allowed active wrist flexion and passive extension to facilitate actuation of the hand. Similarly, the trans-radial simulators incorporated similar features to the trans-radial prosthesis, with the addition of a handle that allowed typically developed children to actuate the device by elbow flexion.

The prostheses and prosthetic simulators were scaled to fit various individuals using techniques from Zuniga and colleagues [34]. The prosthetic devices used in the present study were body-powered, manually adjustable through a BOA dial tensioner system, and were produced primarily using 3D printers. The simulators were produced to mimic partial hand or trans-radial prosthetic devices, which utilize a simple hinge joint in the wrist or elbow (Figure 1). The devices were equipped with voluntary close (VC) terminal devices that resemble hands. Specifically, flexion of the elbow or wrist produced flexion of all five fingers at the metacarpophalangeal joint and the proximal interphalangeal joints. The devices were tensioned to ensure that the index finger and thumb would meet to form a pinch grip. Use of the BOA dial system allowed the participants to actively change the amount of elbow or wrist flexion necessary to flex the phalanges of the device. This VC system allowed for precise manipulation of the prosthetic pinch grip’s strength. Silicone finger pads were added to each finger to supplement traction and enhance pliability for grasping activities.

### 2.4. Functional Near-Infrared Spectroscopy

The hemodynamic response of the contralateral (right) hemisphere of the task (left) limb was measured during all resting and task periods using a continuous wave 24-channel fNIRS system (Hitachi ETG-4000, Hitachi Medical Corporation, Tokyo, Japan). Data were sampled at 10Hz using 690 and 830 nm wavelengths. The cap held eight sources and eight detectors, separated by 3 cm and positioned on the head following the 10–20 international system [35]. A 0.01 Hz high-pass filter and a 0.5 Hz low-pass filter were applied during collection to filter out cardiac noise and low frequency drift [36,37]. A differential path length correction of 6 and a partial volume correction of 1/60 were used. The path length correction was used to correct for the scattering of light through the tissue, while the partial volume correction accounted for the portion of this path that was in the volume of interest, the cortex [38,39]. 

The participants’ head circumferences were taken by measuring around the head at the height of the glabella and the external occipital protuberance. The cap was equipped with adjustable straps to ensure that the cap fit snugly and ensured that the optodes rested against the scalp and could not easily be moved. Additional measurements were taken to align the cap to the center of the scalp. Sagittal measurements were taken from the glabella to the external occipital protuberance, and coronal measurements were taken from the left pre-auricular to the right pre-auricular points.

The AnalyzIR Toolbox [40] was used to analyze the fNIRS data. The AnalyzIR toolbox is a MATLAB (MathWorks, Inc., Natick, MA, USA)-based statistical and visualization package that uses a general linear model (GLM) to determine the activation within the brain. The GLM follows the equation y = Xß + ε, where y is the measurement vector, which contains the discrete time series of measurements of optical absorption for each wavelength. X corresponds to the design matrix, which encodes the timing of stimulus events (rest, task). The variable ß is a column vector of regression coefficients that describe the unknowns in the model and how well the measurement vector fits to the canonical hemodynamic response function. The variable ε defines the measurement error, which is greatly reduced by the auto-regressive iterative least squares (AR-IRLS) method of the GLM implemented by the AnalyzIR toolbox [40,41,42]. These ß regression coefficients can be used to test the null hypothesis that the activation response to the task is zero or can be used to contrast responses between rest and task events.

**Figure 2:**
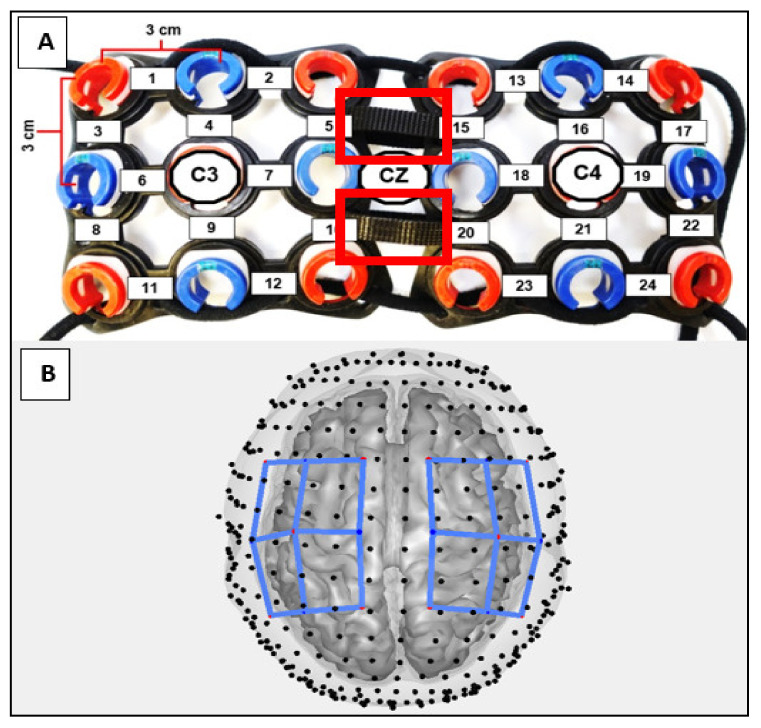
Adjustable headgear and channel arrangement. (**A**) Vertex (Cz) and lateral channels placed over the C3 and C4 motor cortex landmarks associated with motor activity of the hand and arm movements. Red rectangles show the adjustable Velcro straps used to accommodate different head sizes. (**B**) Visualization of headgear after virtual registration to subject brain model. Blue lines indicate placement of the headgear over the brain. M1 was defined as channels: (3–10,15–22). SMA was defined as channels: (1–5,13–17). S1 was defined as channels: (8–12,20–24).

The fNIRS probes were first oriented using a set of landmarks defined in two-dimensional 10–20 coordinates (Figure 2). Using these 10–20 coordinates, the probes were registered to the Colin27 atlas [43], which creates a layered head model that is resized based on the measured circumference of the participant. Regions of interest were defined to inspect more localized brain activity. These regions were: M1, SMA, and S1. The source-detector channels of the three-dimensionally registered probes were assigned weights based on their proximity to the anatomical region of interest, and thus expected relative sensitivity [40]. These weighted regions of interest were based on the optical forward model, which defines the sensitivity of channel-space measurements relative to changes occurring in brain space. These changes in brain space are estimated through simulation of the diffusion of light through tissue [40,44].

### 2.5. Statistical Analysis

To determine any differences in motor performance during prosthetic and simulator use, t-tests were performed on Box and Block scores between groups. Comparisons of Box and Block scores with and without the simulator were also conducted using t-tests. Additionally, a 2 x 3 mixed ANOVA (group (TD, ULR) x ROI (M1, SMA, and S1)) was performed to measure the effects of the prosthesis and simulator use on contralateral activation. To assess the effect of using a prosthetic simulator in TD populations, a repeated-measures ANOVA (Hand (Non-preferred, Simulator) × ROI (M1, SMA, and S1)) was conducted. Prior to running the ANOVAs, Shapiro–Wilk and Levene’s inspections were conducted to assess normality and homogeneity of variance, respectively. Simple effect tests were performed to further assess any interaction effects. An alpha value of 0.05 was considered statistically significant for all tests. All statistical analyses were conducted in R [45].

## 3. Results

### 3.1. Box and Block Scores

The Box and Block test scores (Table 2) between groups using their devices (prosthetic and simulator) did not differ (ULR: 6.0 ± 3.0, TD: 9.0 ± 4.6, *p* = 0.26). The use of the simulator significantly reduced the TD group’s performance (Non-Preferred: 52.2 ± 17.3, Sim: 9.0 ± 4.6, *p* = 0.003).

### 3.2. Contralateral Activations in the ULR Group Compared to the TD Group

**Figure 3:**
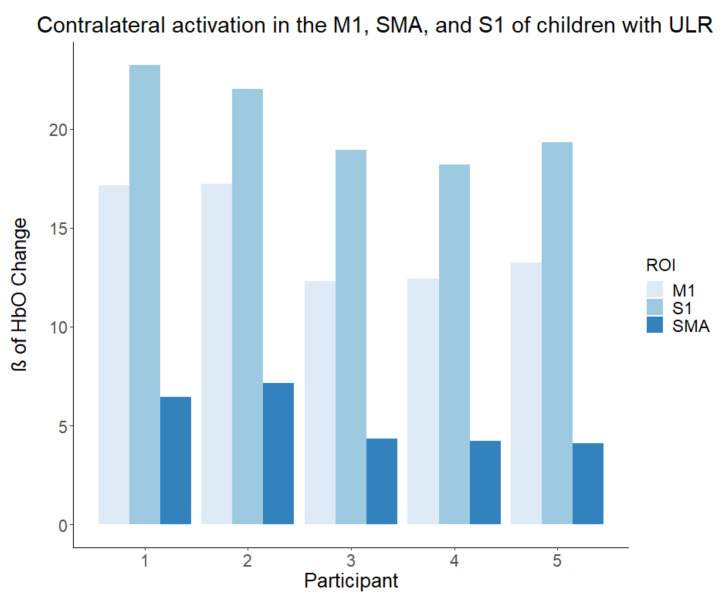
Activation in the ULR group during prosthetic usage. Beta (ß) values produced by the GLM are used as markers of activation with the brain, indicating that the activation matched the expected canonical hemodynamic response. The ULR group displayed lower activation in the M1 and SMA, and comparable S1 activation compared to TD children using the simulator.

**Figure 4:**
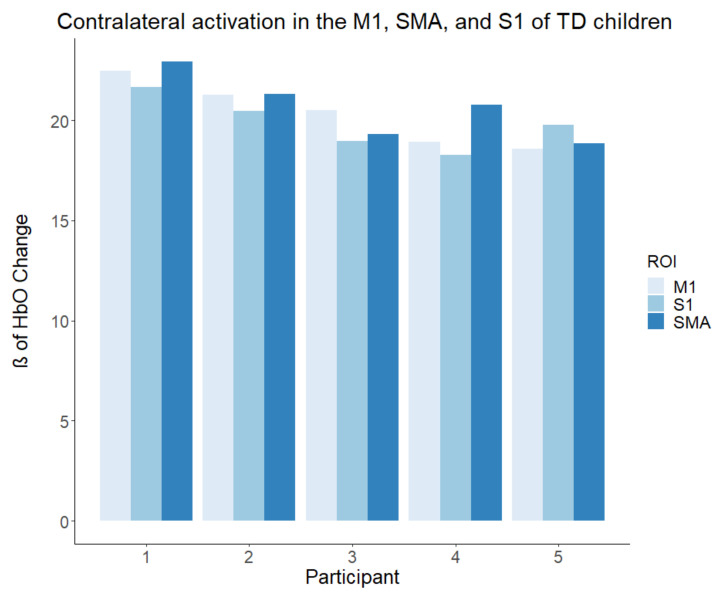
Activation in the TD group using the prosthetic simulator. Beta (ß) values produced by the GLM are used as markers of activation with the brain, indicating that the activation matched the expected canonical hemodynamic response. All TD participants demonstrated higher motor activity compared to the ULR match.

To assess group differences (ULR, TD) in activation within different brain ROIs (M1, SMA, and S1), we conducted a 2 x 3 mixed ANOVA. The ANOVA revealed significant main effects of group (F (1, 8) = 40.46, *p* < 0.001, partial ω2 = 0.80) as well as ROI (F (1.8, 14.3) = 230.25, *p* < 0. 001, partial ω2 = 0.74), which suggested large effect sizes. Additionally, the interaction of these effects reached statistical significance (F (1, 8, 14.3) = 282.57, *p* < 0.001, partial ω2 = 0.78), which also suggested a large effect size. To further understand the interaction observed, simple effects tests were conducted at each ROI. Simple effect tests showed that activation within M1 within the ULR group was lower than the corresponding activation within the TD group (F (1, 8) = 19.441, *p* = 0.002, g = 1.57). Within the SMA, activation was much lower in the ULR group compared to the TD group (F (1, 8) = 251.37, *p* < 0.001, g = 5.63). No differences were observed between groups within the S1 (F (1, 8) = 0.1962, *p* = 0.67, g = −0.16). Results for all participants can be found in Figure 3 and Figure 4.

### 3.3. Contralateral Activations in the TD Group with and without a Simulator

**Figure 5:**
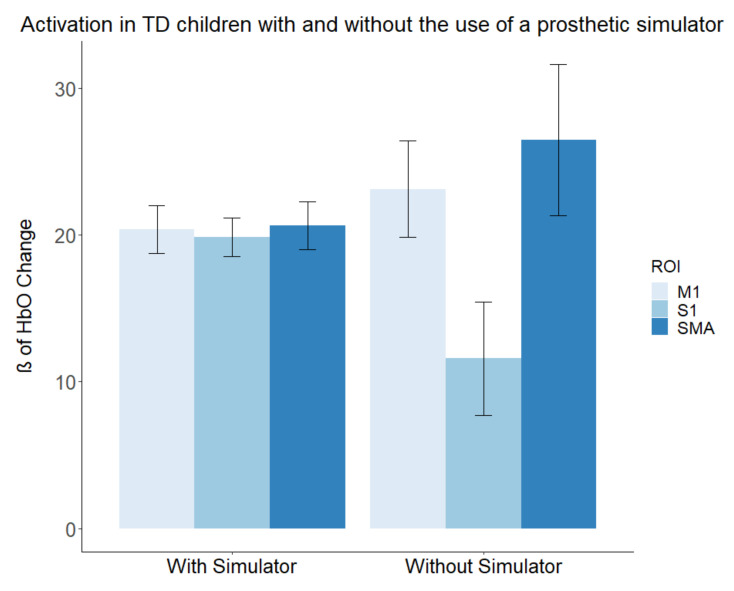
Activation in TD children with and without the prosthetic simulator. Beta (ß) values produced by the GLM are used as markers of activation with the brain, indicating that the activation matched the expected canonical hemodynamic response. The TD group displayed increased S1 activation during the simulator condition.

To assess differences in activation within different brain ROIs (M1, SMA, and S1) produced by tool use (non-preferred hand, prosthetic simulator) within the TD group, we conducted a 2 × 3 repeated-measures ANOVA. We found that the main effect of ROI reached statistical significance (F (1.2, 4.7) = 131.90, *p* < 0.001, partial ω2 = 0.60), indicating a large effect. The main effect of tool use (F (1, 4) = 0.0067, *p* = 0.93, partial ω2 = −0.05) did not reach statistical significance. However, an interaction was observed (F (1.1, 4.6) = 228.58, *p* < 0.001, partial ω2 = 0.56), indicating a large effect. Simple effects tests were conducted to further probe the interaction. Activation within the SMA was significantly higher during the use of the non-preferred hand (F (1, 4) = 12.08, *p* = 0.03, g = 1.24). Alternatively, S1 showed a significant reduction in activation during use of the non-preferred hand compared to the simulator (F (1, 4) = 38.339, *p* = 0.003, g = −2.21). The activation in M1 showed slight increases during the use of the non-preferred hand, but this did not reach statistical significance (F (1, 4) = 6.98, *p* = 0.057, g = 0.94). Mean results of the with- and without-simulator conditions can be found in Figure 5.

## 4. Discussion

The main finding of this study was that use of prosthetic simulators in TD children resulted in greater activation in M1 and SMA, but similar S1 activation, when compared to prosthesis use in the ULR group, who had not used any prosthetic device within the past six months. The higher M1 and SMA activation in the TD group when compared to the ULR group may be further evidence that the experiential motor repertoires that correspond to gross upper limb dexterity are underdeveloped within children with ULR. These results follow the findings of previous studies showing reductions in activation and excitability in the cortex that corresponds to the affected limb [9,46]. Furthermore, it was also found that when the TD group used a prosthetic simulator to perform a gross dexterity task compared to their non-preferred hand, it resulted in significantly lower levels of activation within SMA, but higher for S1, with no significant differences in M1. When comparing differences between the use of the non-preferred hand and the simulator, the increased S1 activation may be in part due to increased proprioceptive and tactile feedback processing.

### 4.1. Group Differences between Prosthesis and Prosthetic Simulator Use

The differences in hemodynamic responses observed in the motor areas between groups reject our hypothesis of the simulator’s ability to emulate the neurological responses in congenital amputees. Indeed, the lower activation observed in the ULR group may be a product of an altered body schema, as suggested by the differences observed in motor cortex activation and excitability [9,46]. Additionally, this idea of altered body schemas is supported by a behavioral study that showed that congenital amputees can determine if images of their affected hand are in unnatural positions faster than images of their non-affected. The authors suggest that this reduction in reaction time is due to the congenital amputee’s ability to skip referencing their body schema [47].

Should the schema be innate to every individual, the reduced behavioral outcomes of the affected limb may be the cause of the observed activation differences. Previous investigations have reported that the motor cortex is subject to structural and plastic changes following traumatic amputation, resulting in reduced gray matter [48,49]. These findings may suggest a progressive degeneration evoked by the loss of afferent input from a missing limb. The creation of strengthened neuronal groups, driven by increased behavior, predicts these results [13]. If children with ULR receive less afferent input from their environment with their affected limb, this may weaken the neuronal groups that correspond to the limb. Our results are in line with the aforementioned studies, showing a lesser response within the motor cortex within the ULR group compared to the TD group. Moreover, the motor activation differences could be due to a reduction in degrees of freedom during the task, as the ULR group is bound to either elbow or wrist active flexion and passive extension to activate the prosthesis’s grasping action (Figure 1). While the partial hand and trans-radial simulators mimic this by forcing the user to actively flex at the wrist or elbow drive flexion of the simulator phalanges, they still must use their fingers to grip the devices.

The insignificant differences observed within S1 during the use of a prosthesis and prosthetic simulator may suggest that the devices provide similar sensory input. Children wearing upper-limb body-powered prostheses rely on socket forces and torques to gather information about the prosthesis and the environment to perform activities of daily living [50]. The use of a simulator may cause shifts in the user’s attention to mechanical feedback. However, NGST suggests that the brain is not neatly partitioned, and perception does not occur in one localized region, which executes a command to perform a motor output. Instead, the motor behavior consequences are a part of the perception, and mappings within the motor and sensory areas are recursively linked [13,51]. Thus, the action of performing a task with a tool such as a prosthesis simulator or a prosthesis may create similar conditions to produce these reentrant perception–action loops.

### 4.2. Differences in Activation Produced by Prosthetic Simulator Use

Use of the simulator in the TD group significantly attenuated activation within the SMA, and only slightly in M1, while activation in S1 significantly increased. These results reject our hypothesis as S1 was the only region to increase in activation from simulator use. Activation in M1 may have been unaffected by the simulator as the children were still utilizing their entire limb. While the simulators function to force the user to adopt new kinematic solutions to perform the task (elbow flexion or wrist flexion for terminal device closure; see Figure 1), the user still had to use their distal effectors to stabilize the device. Furthermore, the neuronal groups associated with the use of the limb have already been selected; selection of motor repertoires more appropriate for simulator use may not be evoked after acute use of the device within M1 [13,52]. Elimination of the fingers’ use may have been a driving factor that led to the decreased SMA activation. By simplifying the action to flexion of either the wrist or elbow, there may have been a reduction in planning needed. Alternatively, the SMA has been noted to assist with rapid, repetitive tasks. In the simulator condition, children were unable to rapidly move blocks from one partition to the other, making each action more deliberate and thus less demanding on the motor system. It should be noted that the TD group exhibited a large amount of variability of activation during the non-preferred hand condition, which may be a product of different development milestones, and has consequently decreased the significance of our observation.

While the mechanical restrictions imposed by the simulator may not be enough to simulate the potentially weakened neuronal groups present in children with ULR, they may be conducive to emulating the afferent feedback utilized by the use of a prosthetic device. Such afferent feedback could be tactile in nature, supplying information about forces applied by the tool and the relation of these forces to the closure of the terminal device [50,53]. The simulator’s use may allow for the exploration of the neural mechanisms used to facilitate the motor control used within body-powered prostheses. As individuals with amputations use their body-powered prosthesis, they must learn how to coordinate reaching and closure of the terminal device [7]. The precision of this closure force is often difficult to attain due to lack of proprioceptive feedback from the device [7]. Use of a prosthetic simulator may allow for the emulation of these conditions, as seen by the increased S1 activation, which may signal an upregulation of tactile and proprioceptive feedback from the proximal limb segments. Thus, prosthetic simulators may prove useful during periods of prosthesis manufacturing for individuals with unilateral upper limb reductions or amputations, allowing them to practice the mechanism of their prosthesis with their non-affected limb through bilateral transfer [6]. Alternatively, future studies may be able to leverage prosthetic simulators to study the proprioceptive feedback mechanisms seen in prosthesis use in a larger cohort of typically developing participants than often seen in prosthetic studies. A longitudinal observation of the effect of practice in both the ULR and TD groups would be informative to determine if the increased S1 activation is simply a product of increased cognitive burden from learning a new tool, or if training alters the somatosensory cortex of TD and ULR children differently.

The limitations of the present investigation are related to the small number of children participating in the study as there were few local pediatric ULR participants. This limited sample size greatly reduces the power of our study and its generalizability to larger populations. The results of this study should thus be viewed as preliminary data. However, to help alleviate this shortcoming, we have made an effort to display each individual’s response (Figure 3 and Figure 4) to allow for the comparison of individual results with respect to the results of the group analyses. Additionally, some participants had prior experience with a prosthetic device, thus reducing the true novelty of the motor tasks. However, all children reported not using their prosthesis for six months or longer before participating in the study. Levels of amputations within the ULR group varied from partial hand to trans-radial. While simulators were made to emulate both levels of amputation, a more homogenous amputation level would eliminate any cofounding effects. A broad age range of children was recruited and introduced variances of motor development. The fNIRS system was also vulnerable to motion-induced artifacts and systemic physiological noise. The physiological noise is produced by heart rate, respiration, and Mayer waves. The pre-filtering utilized reduces the noise produced by the cardiac cycle and Mayer waves, but respiration often falls within the same frequency of the hemodynamic response. The AR-IRLS algorithm of the GLM is well suited to deal with both sources of noise through pre-whitening of the data [41]. We suggest that future studies should address the forementioned limitations by recruiting larger samples of homogenous participants and utilizing multiple methodologies of brain imaging, such as both fNIRS and fMRI. Moreover, we suggest examining these activation patterns over time with training to determine if the simulator’s ability to elicit increased S1 activation is a lasting effect.

## 5. Conclusions

In conclusion, use of a prosthesis simulator in typically developing populations leads to decreased activation in the SMA, with no change in M1 and an increase in activation in S1. Compared to the ULR group, children using the simulator exhibited much more activation within their motor areas (SMA and M1), while there was no observable difference in S1 activation. These results suggest that simulators cannot emulate the neurophysiological differences seen in the ULR group by mechanically restricting the limbs. However, prosthetic simulators may provide individuals with limb reductions and typically developing children the ability to simulate the need for increased tactile and proprioceptive feedback when performing motor tasks. This simulation may be leveraged by future studies utilizing larger cohorts of typically developing individuals to determine if training paradigms that focus on the tactile and proprioceptive feedback from the device significantly improve performance compared to traditional training paradigms. If so, these feedback-focused paradigms may prove beneficial by allowing individuals with unilateral limb reductions to practice with either a simulator or their actual prosthesis to improve prosthetic functionality and reduce prosthetic rejection rates.

## Figures and Tables

**Table 1:** Participant characteristics (*n* = 10).

Experimental Group (Congenital Upper Limb Reduction)						
ID	Sex	Age (Years)	Preferred Side	Reduction Level	Affected Side	Ability to Pinch
1	Female	6.2	Right	Partial Hand	Left	No
2	Female	8.2	Right	Trans-Radial	Left	No
3	Male	11.1	Right	Partial Hand	Left	No
4	Male	5.1	Right	Trans-Radial	Left	No
5	Male	13.2	Right	Partial Hand	Left	No
M ± SD		8.76 ± 3.37				
**TD Group (Typically Developing)**						
1	Female	6.4	Right	None	None	Yes
2	Female	8.3	Right	None	None	Yes
3	Male	11.3	Right	None	None	Yes
4	Male	5.6	Right	None	None	Yes
5	Male	13.2	Right	None	None	Yes
M ± SD		8.96 ± 3.23				

**Table 2:** The Box and Block score is determined by how many blocks can be moved to one side of a partitioned box to another within 1 min. Children with ULR performed the task with their non-affected limb and their prosthesis, while TD children performed the task with their non-dominant limb, with and without the simulator.

Box and Block Score					
ULR Group			TD Group		
Participant	Preferred Limb	Prosthesis/Simulator	Participant	Non-Preferred Limb	Prosthesis/Simulator
1	37	3	1	37	6
2	31	3	2	30	6
3	50	7	3	65	17
4	39	7	4	62	9
5	42	10	5	67	7
Mean	39.80	6	Mean	52.2	9
SD	6.98	3	SD	17.34	4.64

## Data Availability

The data presented in this article are available upon request from the corresponding author. The data are not publicly available due to privacy concerns.
